# PD-L1, STAT3, IL6, and EGFR Immunoexpressions in High-Grade Osteosarcoma

**DOI:** 10.1155/2024/9036225

**Published:** 2024-02-23

**Authors:** Nayla Rahmadiani, Eviana Norahmawati, Agustina Tri Endharti, Ailen Oktaviana Hambalie, Satria Pandu Persada Isma

**Affiliations:** ^1^Department of Anatomical Pathology, Faculty of Medicine Universitas Brawijaya, Saiful Anwar General Hospital, Malang, Indonesia; ^2^Department of Biomedical Sciences, Faculty of Medicine Universitas Brawijaya, Malang, Indonesia; ^3^Department of Orthopaedics and Traumatology, Faculty of Medicine Universitas Brawijaya, Saiful Anwar General Hospital, Malang, Indonesia

## Abstract

**Introduction:**

Immunotherapy has been widely used in the treatment of various malignancies with satisfactory results. One of the agents for immunotherapy is an inhibitor of programmed cell death-1 and its ligands (PD-1 and PD-L1). However, attempts at utilizing PD-1/PD-L1 immunotherapy in osteosarcoma have not yielded favorable results. This may be due to differences in PD-L1 regulation and the immune landscape in osteosarcoma, as the mechanism is still poorly understood. Therefore, elucidating PD-L1 regulation in osteosarcoma is paramount in order to improve treatment results using immunotherapy.

**Methods:**

This is a cross-sectional study conducted in the Department of Anatomical Pathology of Saiful Anwar Hospital using 33 paraffin blocks of confirmed cases of osteosarcoma. Immunohistochemical staining using PD-L1, STAT3, IL6, and EGFR was performed. Statistical analyses were subsequently performed on the immunoexpression data of these antibodies.

**Results:**

PD-L1, STAT3, IL6, and EGFR expressions were found in 6 (18.2%), 6 (18.2%), 28 (84.8%), and 30 (90.9%) cases, respectively. There were significant correlations between PD-L1 and STAT3 (*r* = 0.620, *p*=<0.001), PD-L1 and EGFR (*r* = 0.449, *p*=0.009), as well as STAT3 and EGFR (*r* = 0.351, *p*=0.045).

**Conclusion:**

The existence of a correlation between PD-L1, STAT3, and EGFR indicates the potential role of STAT3 and EGFR in PD-L1 regulation in osteosarcoma, which may become the basis for targeted therapy.

## 1. Introduction

Sarcoma of bone is a rare neoplasm with various histological subtypes. These sarcomas account for <1% of cancers in adults and 7–15% of malignancies in the pediatric population [1]. Osteosarcoma is the most common nonhaematological primary bone malignancy [1, 2]. Current therapeutic modalities result in a 5-year survival rate of 50–70%; however, this rate tends to be stagnant. Furthermore, the variation in response to therapy, the presence of metastasis, and recurrence may result in a much lower survival rate. Thus, new therapeutic modalities which can improve the prognosis of osteosarcoma patients are much needed [1–3].

One therapeutic modality that has recently been widely researched and provides quite promising results in various types of cancer is immunotherapy [1–3]. Immune checkpoint molecules such as programmed cell death-1 (PD-1) and programmed cell death ligand-1 (PD-L1) play a role in the immune tolerance process by inhibiting excessive T cell immune responses. Osteosarcoma cells express PD-L1, and this affects their ability to evade immune surveillance [1, 4, 5].

However, in contrast to several other cancers which showed satisfactory results, the results of immunotherapy targeting the PD-1/PD-L1 axis in osteosarcoma have been unsatisfactory [6]. This may be due to differences in PD-1/PD-L1 regulation and the immune landscape in osteosarcoma which is also often referred to as a “cold tumor” [7]. However, numerous literature have shown a significant association between PD-L1 expression and a poor prognosis in osteosarcoma. In addition, there have been efforts to combine more than one immune checkpoint inhibitor (ICIs) or modulation of the tumor microenvironment (TME) to increase the effectiveness of therapy [1, 7–9].

PD-L1 regulation itself is a complex process that is influenced by various factors: genomic changes, epigenetic modifications, transcriptional regulation, posttranscriptional modifications, and posttranslational modifications. In various cancers, an association between signal transducer and activator of transcription 3 (STAT3), interleukin 6 (IL6), and epidermal growth factor receptor (EGFR) on PD-L1 expression has been shown in varying degrees [10]. The literature states that the regulatory mechanism of PD-L1 in osteosarcoma is still unclear, so it is important to study the regulatory mechanism of PD-L1 in osteosarcoma to direct future therapy [7]. There have been some studies which revealed the involvement of IL6 and STAT 3 in increasing PD-L1 expression in osteosarcoma [5, 11–13]. Moreover, the EGFR pathway is generally known to be one of the pathways which can increase PD-L1 expression in various cancers, although there have been no studies regarding this specifically in osteosarcoma [10, 14, 15]. Up until now, there have been no studies examining the relationship between IL6, EGFR, and STAT3 and PD-L1 using immunohistochemical expression in osteosarcoma patient subjects. Furthermore, there are still no studies examining whether there are differences in the PD-1/PD-L1 regulation pathways in pediatric and nonpediatric osteosarcoma patients, although there are studies that state that there may be differences in sarcoma behavior between adult cases and children. This may influence immunotherapy strategies in pediatric and nonpediatric patients [16].

This study aims to determine the correlation between immunoexpression of STAT3, IL6, and EGFR and immunoexpression of PD-L1 in osteosarcoma patients, while also evaluating their expressions in pediatric and nonpediatric patients. This research is expected to provide additional knowledge regarding the mechanisms of PD-L1 regulation in osteosarcoma, in the hope of becoming a catalyst for further research on this topic and the basis for anti-PD-L1 combination therapy with other targets involved in PD-L1 regulation, with the end goal of yielding better outcomes for the patients.

## 2. Methods

### 2.1. Study Population

This is a cross-sectional study conducted in the Department of Anatomical Pathology of Saiful Anwar Hospital using 33 paraffin blocks of confirmed cases of osteosarcoma which fulfill the inclusion criteria using a total sampling method. To minimize bias, we limit the osteosarcoma to being of high grade only. Based on the latest World Health Organization classification, this includes conventional osteosarcoma, telangiectatic osteosarcoma, small-cell osteosarcoma, and high-grade surface osteosarcoma [17]. The inclusion criteria were as follows: (1) paraffin blocks of confirmed cases of osteosarcoma treated at the Orthopedics and Traumatology Department of Saiful Anwar Hospital during the period of January 2019 to September 2023; and (2) the slide produced from the paraffin block must contain at least 100 tumor cells. Meanwhile, the exclusion criteria were as follows: (1) damaged or missing paraffin block; and (2) paraffin blocks with insufficient tissue for further immunostaining; and (3) cases with only decalcification specimens available. The general characteristics of the patients were retrieved from the medical records, including gender, age, and tumor location. Age was further classified into pediatric and nonpediatric based on the cut-off determined by the American Academy of Pediatrics of ≤21 years old [18].

### 2.2. Immunohistochemical Staining and Interpretation

Paraffin blocks were sectioned using microtome with 4-5 *μ*m thickness, mounted onto glass slides, and subsequently stained according to the method described by Magaki et al. [19]. The antibodies used for staining were as follows: (1) Monoclonal mouse antihuman PD-L1 clone 22c3 (Dako Agilent Technologies Inc, Santa Clara, CA) with 1 : 100 dilution; (2) monoclonal mouse antihuman STAT3 (GeneTex Inc, Irvine, CA) with 1 : 50 dilution; (3) Polyclonal rabbit antihuman IL6 (GeneTex Inc, Irvine, CA) with 1 : 400 dilution; and (4) polyclonal rabbit antihuman EGFR (GeneTex Inc, Irvine, CA) with 1 : 200 dilution.

The intensity of the staining and positivity of tumor cells after PD-L1, STAT3, IL6, and EGFR immunostaining were evaluated using a light microscope at 400x magnification on at least 100 tumor cells for every slide. PD-L1 immunoexpression was assessed using the tumor proportion score (TPS).

(1) $$\begin{array}{c} \text{TPS}=\frac{\text{PDL}1\;\text{positive}\;\text{tumor}\;\text{cells}}{\text{viable}\;\text{tumor}\;\text{cells}}\times 100. \end{array}$$

PD-L1 is interpreted as negative if there is no partial or complete staining of any intensity on the cell membrane in <1% of viable tumor cells, and positive for PD-L1 expression if there is partial or complete staining of any intensity on the cell membrane in ≥1% of viable tumor cells. STAT3, IL6, and EGFR interpretations were carried out by assessing the intensity score and positivity score. Intensity is scored as follows: 0 (negative), 1 (weakly positive), 2 (moderately positive), and 3 (strongly positive). Tumor cell positivity score in STAT3 and IL6 was scored as: 0 (negative), 1 (1–50%), 2 (51–75%), and 3 (>75%). Meanwhile, the tumor cell positivity score in EGFR was scored as: 0 (negative), 1 (<25%), 2 (25–75%), and 3 (>75%) [20–24]. The total score (product of intensity and positivity scores) of ≤2 for STAT3 and EGFR is deemed as low expression and >2 as high expression, while a total score of ≤3 for IL6 is regarded as low expression and >3 as high expression. Interpretation was performed by two different pathologists, both blinded to the clinical data of the patients.

### 2.3. Statistical Analysis

Descriptive analysis was performed on patients' general data and immunoreactivity and was presented as frequencies and percentages or means and standard deviations, according to the type of variable (categorical vs. continuous). Normality tests were performed using the Shapiro–Wilk test on the age and immunoreactivity of all examined antibodies. Chi-square tests were performed to evaluate the difference between age and immunoreactivity of PD-L1, STAT3, IL6, and EGFR. Spearman correlation tests were performed to evaluate the correlation between age and immunoreactivity of the four antibodies, as well as correlation among the four antibodies with each other. Additional chi-square and Fisher exact tests were also performed to evaluate the difference in immunoexpressions of PDL-1 vs STAT3, IL6, and EGFR. All statistical analyses were performed using SPSS v.20 (IBM SPSS, Armonk, NY).

## 3. Results and Discussion

### 3.1. Characteristics of Osteosarcoma Patients

33 paraffin blocks from osteosarcoma patients were included in this study, which were predominated by male patients (63.6%). The patients' ages ranged from 8 to 59 years old, with a mean age of 27.48 ± 15.22 years old and a median age of 24 years old. Nonpediatric patients were slightly higher in number than pediatric patients (18 vs 15, respectively). The femur was the most common site for osteosarcoma in our study.  Table 1 details the overall characteristics of the study population. The characteristics of our study population were in accordance with the literature which stated that osteosarcoma tends to have a male predominance and that the age may range from children to older adults. The femur was also mentioned in the literature as one of the most common sites for osteosarcoma, along with the tibia and humerus [17].

### 3.2. Immunoexpressions of PD-L1, STAT3, IL6, and EGFR in Osteosarcoma

**Figure 1:**
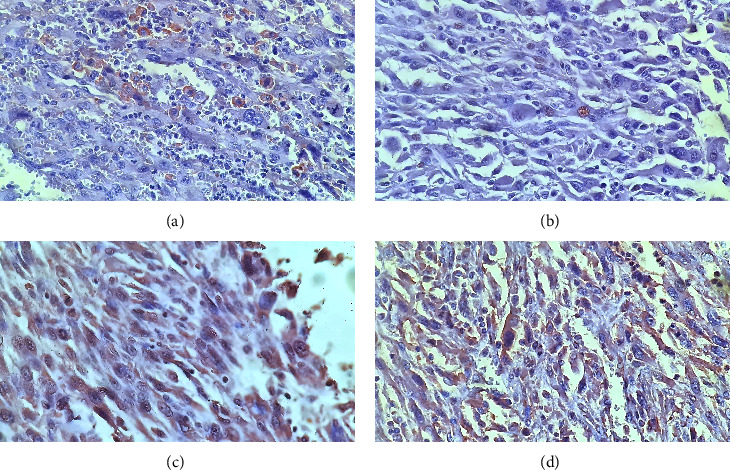
Positive immunoexpressions of PD-L1, STAT3, IL6, and EGFR. Upon examination using a light microscope (400x magnification), PD-L1 showed membrane staining (a), while STAT3 showed nuclear staining (b). Membranous and cytoplasmic staining of the tumor cells were found in IL6 (c) and EGFR (d).

Immunoexpressions of PD-L1, STAT3, IL6, and EGFR were found in 6/33 (18.2%), 6/33 (18.2%), 28/33 (84.8%), and 30/33 (90.9%) cases, respectively (Table 1, Figure 1).  Table 2 presents the overall immunoreactivity of all antibodies in the study population. Statistical analysis using Spearman correlation revealed that there were significant correlations between PD-L1 and STAT3 (*r* = 0.620, *p* < 0.001), PD-L1 and EGFR (*r* = 0.449, *p*=0.009), as well as STAT3 and EGFR (*r* = 0.351, *p*=0.045). Meanwhile, there is no correlation between PD-L1 and IL6, STAT3 and IL6, and EGFR and IL6.

Upon further statistical analysis using chi-square and Fisher exact tests between PD-L1 immunoexpression vs STAT3, IL6, and EGFR, significant differences were found between PD-L1 and STAT3 (*p*=0.005), as well as PD-L1 and EGFR (*p*=0.049). However, no significant difference was found between PD-L1 and IL6 (*p*=0.627) (Table 3).

In general, PD-L1 regulation is a complex process influenced by genomic changes, epigenetic modifications, transcriptional regulation, posttranscriptional modifications, and posttranslational modifications [10]. PD-L1 can be influenced by transcriptional regulation such as inflammatory signaling related to Interferon (IFN) and IL6, as well as oncogenic signaling related to EGFR [10, 14]. IL6 can induce PD-L1 expression via the IL6-JAK-STAT3 pathway. EGFR mutations can increase PD-L1 expression; clinically, this phenomenon can be seen in the disruption of PD-L1 expression after therapy with EGFR tyrosine kinase inhibitors (TKI) [7, 8, 10–15].

Studies have revealed that PD-L1 expression across sarcoma types seems to vary. However, the expression of this molecule in sarcoma becomes the basis of immunotherapy research to develop alternative treatments for sarcoma [25–27]. Furthermore, a recent meta-analysis showed that PD-L1 overexpression in bone and soft tissue sarcoma could predict poor overall survival, metastasis-free survival, and event-free survival [1]. The overexpression of PD-L1 was correlated with a higher rate of tumor metastasis, a more advanced tumor grade, and more T lymphocyte infiltration [27]. This highlights the importance of the PD-1/PD-L1 axis in sarcoma. PD-L1 expression was significantly increased in bone tumors, with malignant and high-grade bone tumors expressing higher levels of PD-L1 [28]. The percentage of PD-L1-positive osteosarcoma varied between studies, ranging from 47% to 62.5% [28, 29].

In osteosarcoma, PD-L1 is overexpressed by tumor cells and cells in the TME and inhibits cytotoxic T cells by binding to the PD-1 receptor on activated T cells, which leads to immune escape and supports tumor growth. The regulation of PD-L1 expression in osteosarcoma is still not completely understood [7]. However, there is a study on osteosarcoma cell lines which stated that there is activation of histone deacetylase 6 (HDAC6) and IL6 which leads to activation of STAT3 in osteosarcoma, which ultimately induces PD-L1 expression. HDAC6 can activate the STAT3 transcription factor and indirectly modulate STAT3 phosphorylation which leads to increased PD-L1 expression in tumor cells. IL6 can activate STAT3 in osteosarcoma and cause an increase in PD-L1 expression in a dose-dependent manner, and this has been proven in vitro [11]. While EGFR was found to be overexpressed in osteosarcoma, there is still no study linking its involvement in the regulation of PD-L1 expression in osteosarcoma [10, 14, 15]. Our findings corroborated the notion that PD-L1 is regulated by STAT3, as shown by the positive correlation between them. The positive correlation between PD-L1 and EGFR indicates the possibility of EGFR playing a role in PD-L1 regulation in osteosarcoma.

The correlation between STAT3 and EGFR may be explained by the fact that EGFR activation will lead to the activation of the phosphatidylinositol-3 kinase (PI3K) and protein kinase B (AKT) pathway, the mitogen-activated protein kinases (MAPK)/extracellular-signal-regulated kinases (ERK) pathways, as well as STAT3, all of which will eventually result in the proliferation, invasion, angiogenesis, and inflammatory responses–the hallmarks of cancer [13, 30, 31]. The presence of a positive correlation between PD-L1, STAT3, and EGFR indicated the possibility of targeting STAT3 and EGFR aside from PD-1/PD-L1 in order to maximize the outcome for the patient, as shown in several studies [30–36].

Upon comparison between age and immunoexpressions of PD-L1, STAT3, IL6, and EGFR, it was found that there were no significant differences (*p*=0.805, *p*=0.249, *p*=0.883, *p*=0.965, respectively) (Table 4). In addition, it was observed that there were no correlations between age and immunoexpressions of PD-L1 (*r* = −0.033, *p*=0.854), STAT3 (*r* = −0.201, *p*=0.262), IL6 (*r* = 0.023, *p*=0.898), and EGFR (*r* = −0.059, *p*=0.745). So far, the literature regarding the difference in immunolandscape between pediatric and nonpediatric osteosarcoma is still scarce. According to one study, the poorer outcome in older patients might be partly explained by the skewing towards immunosuppressive phenotype in older patients. Osteosarcoma in older adults tends to have higher immune infiltration but also have higher PD-L1 expression and lower B-cell abundance [37]. Another study mentioned that pediatric bone sarcomas tend to have lower immunogenicity [6].

The limitation of this study is the small number of samples. Another limitation is the nature of immunohistochemistry examination for PD-L1, as there are factors which may influence the result, such as tissue sampling (biopsy vs resection, primary site vs metastasis site), paraffin block storage, the type of clone used and various manufacturers, protocols used, and scoring method. Moreover, gene expression might differ from protein expression [27, 38–41]. It is important to interpret the results presented in this study within the context of the study population and the methodology used (i.e., only examining osteosarcoma of high grade, using PD-L1 clone 22c3, and using paraffin blocks stored in our department within the range of 2019–2023). Future confirmatory, large-scale studies comparing the gene and protein expression of PD-L1 are needed. Studies regarding the combined use of anti-PD-1/PD-L1 with anti-STAT3 and/or EGFR are warranted.

## 4. Conclusion

This study revealed the correlation between PD-L1, STAT3, and EGFR, which may indicate the role of STAT3 and EGFR in PD-L1 regulation in osteosarcoma. This may become the basis of further research targeting STAT3 and EGFR in addition to PD-1/PD-L1 immunotherapy in osteosarcoma.

## Figures and Tables

**Table 1:** Characteristics of study population.

Characteristics	Frequency	Percentage (%)
Age group		
Pediatric	15	45.5
Nonpediatric	18	54.5
Gender		
Male	21	63.6
Female	12	36.4
Location		
Humerus	1	3.0
Maxilla	1	3.0
Tibia	11	33.3
Femur	15	45.5
Mandibula	4	12.1
Cranial	1	3.0

**Table 2:** Overall PD-L1, STAT3, IL6, and EGFR immunoexpressions in study population.

Immunoexpressions	Frequency	Percentage (%)
PD-L1		
Negative	27	81.8
Positive	6	18.2
STAT3		
Negative	27	81.8
Low expression	6	18.2
IL6		
Negative	3	9.1
Low expression	7	21.2
High expression	23	69.7
EGFR		
Negative	5	15.2
Low expression	15	45.4
High expression	13	39.4

**Table 3:** Crosstabulation between PD-L1, STAT3, IL6, and EGFR immunoexpressions.

	PD-L1 immunoexpression				*p* value (using chi-square test)
	Negative		Positive		
	*n*	%	*n*	%	
Immunoexpression of STAT3					
Negative	25	92.6	2	33.3	0.005 (with Fisher exact test)
Low expression	2	7.4	4	66.7	
Immunoexpression of EGFR					
Negative	5	18.5	0	0.0	0.049
Low expression	14	51.9	1	16.7	
High expression	8	29.6	5	83.3	
Immunoexpression of IL6					
Negative	3	11.1	0	0.0	0.627
Low expression	6	22.2	1	16.7	
High expression	18	66.7	5	83.3	

**Table 4:** PD-L1, STAT3, IL6, and EGFR immunoexpressions in pediatric and nonpediatric osteosarcoma.

Immunoexpression interpretations	Pediatric		Nonpediatric		*p* value
	*n*	%	*n*	%	
PD-L1					
Negative	12	80.0	15	83.3	0.805
Positive	3	20.0	3	16.7	
STAT3					
Negative	11	73.3	16	88.9	0.249
Low expression	4	26.7	2	11.1	
IL6					
Negative	1	6.7	2	11.1	0.883
Low expression	3	20.0	4	22.2	
High expression	11	73.3	12	66.7	
EGFR					
Negative	2	13.3	3	16.7	0.965
Low expression	7	46.7	8	44.4	
High expression	6	40.0	7	38.9	

## Data Availability

The data used to support the findings of this study are available from the corresponding author upon reasonable request.
